# Membrane Vesicles Are the Dominant Structural Components of Ceftazidime-Induced Biofilm Formation in an Oxacillin-Sensitive MRSA

**DOI:** 10.3389/fmicb.2019.00571

**Published:** 2019-03-21

**Authors:** Xinlong He, Shuang Li, Yi Yin, Jiahui Xu, Weijuan Gong, Guocai Li, Li Qian, Yinyan Yin, Xiaoqin He, Tingting Guo, Yuzheng Huang, Feng Lu, Jun Cao

**Affiliations:** ^1^Institute of Translational Medicine, Medical College, Yangzhou University, Yangzhou, China; ^2^NHC Key Laboratory of Parasitic Disease Control and Prevention – Jiangsu Provincial Key Laboratory on Parasite and Vector Control Technology, Jiangsu Institute of Parasitic Diseases, Wuxi, China; ^3^Jiangsu Co-innovation Center for the Prevention and Control of Important Animal Infectious Diseases and Zoonoses, College of Veterinary Medicine, Yangzhou University, Yangzhou, China; ^4^The Third People’s Hospital of Wuxi, Wuxi, China; ^5^Jiangsu Key Laboratory of Integrated Traditional Chinese and Western Medicine for Prevention and Treatment of Senile Diseases, Yangzhou University, Yangzhou, China; ^6^Center for Global Health, School of Public Health, Nanjing Medical University, Nanjing, China; ^7^Public Health Research Center, Jiangnan University, Wuxi, China

**Keywords:** methicillin-resistant *Staphylococcus aureus*, MRSA, antibiotic resistance, biofilm, membrane vesicle

## Abstract

Methicillin-resistant *Staphylococcus aureus* (MRSA) has received increasing attention in recent years. However, the characteristics and relevant mechanisms of biofilm formation in oxacillin-sensitive MRSA (OS-MRSA) are poorly understood. This study was designed to characterize biofilm formation in OS-MRSA BWSA15 in response to ceftazidime (TZ) by comparing the methicillin-sensitive *S. aureus* (MSSA) strain BWSA23 and the oxacillin-resistant MRSA (OR-MRSA) strain BWSA11. The biofilms and biofilm-forming cells were observed by electron microscopy. Biofilms grown on microtiter plates were chemically decomposed and analyzed by Fourier transform infrared spectroscopy. The transcriptional regulation of genes associated with methicillin resistance, surface adhesion, fatty acid biosynthesis, and global regulation (sigma B) was investigated. A significant increase in biofilm formation ability (10.21-fold) and aggregation ability (2.56-fold) was observed in BWSA15 upon the treatment with TZ (16 μg/ml). The TZ-induced biofilm formation in BWSA15 was characterized by a disappearance of polysaccharide-like extracellular substances and an appearance of a large number of intercellular MVs from extracellular matrix. Few MVs were identified in the biofilms formed by BWSA11 and BWSA23. There was a significant upregulation of *mecA, sigB*, and fatty acid biosynthesis-associated genes and downregulation of *icaA, icaD, clfA, clfB*, and *fnaA* in BWSA15 upon the treatment with TZ. The formation of intracellular junctions of MVs in the biofilms of BWSA15 was mediated by a significant increase in the proportion of proteins as well as by an increase in the proportion of non-ionized carboxyl groups in fatty acids. This study demonstrated that beta-lactam antibiotics can induce biofilm formation in OS-MRSA, and the biofilm induction in OS-MRSA can mainly be attributed to exposed MVs with increased hydrophobicity rather than polysaccharide intercellular adhesins, cell wall-anchored surface proteins, and extracellular DNA.

## Introduction

*Staphylococcus aureus*, a pathogenic bacteria, can cause various infections, including skin and soft tissue infections, endocarditis, pneumonia, and even septicemia (Tong et al., 2015). Methicillin has been used to effectively control infections caused by β-lactamase-producing *S. aureus*. However, methicillin-resistant *S. aureus* (MRSA) soon appeared due to the use of methicillin in clinical settings (Jevons, 1961). Currently, MRSA is one of the most common pathogens that causes nosocomial infections worldwide (den Heijer et al., 2013; Udobi et al., 2013; Chen and Huang, 2014). Among these clinical MRSA strains, oxacillin-sensitive MRSA (OS-MRSA) has received increasing attention due to the latent but highly inducible methicillin resistance exhibited by this strain (Hososaka et al., 2007; Pu et al., 2014; Chung et al., 2016).

In recent years, MRSA has also received attention because these bacteria have been found to form biofilms that generally cause chronic infections (Cha et al., 2013). Many studies have demonstrated that the physicochemical properties of biofilms formed by MRSA are different from those formed by methicillin-sensitive *S. aureus* (MSSA) (Atshan et al., 2012a; Ohadian Moghadam et al., 2014), and this difference has been associated withthe acquisition of the *mecA* gene. Studies have also shown that the biofilm formation ability of clinically derived MRSA is positively correlated with *mecA* expression (Cortes et al., 2015), suggesting that there are differences in biofilm regulatory pathways or regulatory levels between OS-MRSA and other MRSA strains. Microbial surface components recognizing adhesive matrix molecules (MSCRAMM) are commonly found in *S. aureus* strains (Atshan et al., 2012b). Among these MSCRAMMs, polysaccharide intercellular adhesins (PIAs) or polymeric N-acetyl-glucosamine (PNAG) and surface protein adhesins are considered to be important substances that contribute to the surface adhesion of MSSA and MRSA (Pozzi et al., 2012; McCarthy et al., 2015), respectively. However, it is unclear whether the differences in the biofilm properties exhibited by MRSA at different expression levels of *mecA* are functionally attributed to the regulation of MSCRAMMs.

*Staphylococcus aureus* strains have been found to liberate membrane vesicles (MVs) during growth *in vitro* and *in viv*o (Lee et al., 2009; Gurung et al., 2011). *S. aureus* MVs have also been demonstrated to play an important role in the delivery of virulence factors to host cells (Gurung et al., 2011; Hong et al., 2011; Jeon et al., 2016). Our recent study indicated that MVs also play a role in surface adhesion and intercellular aggregation during MRSA biofilm formation (He et al., 2017). However, the molecular mechanism underlying the involvement of MVs in biofilm formation remains poorly understood. Ceftazidime (TZ) belongs to the cephalosporin class of beta-lactams, which has been widely used in clinical settings. This study was mainly designed to investigate the phenotypic characteristics of OS-MRSA in a simulated clinical environment, which is a therapeutically non-effective dose of TZ, and to further elucidate the roles of MSCRAMMs and MVs in the regulation of biofilm formation.

## Materials and Methods

### Bacterial Strains and Growth Conditions

Three *S. aureus* clinical isolates obtained from the burn wounds of different burn patients in the Third People’s Hospital of Wuxi from 2015 to 2016, namely, BWSA11, BWSA15, and BWSA23, were used in this study. This study was approved by the Ethics Committees of the Third People’s Hospital of Wuxi and Jiangsu Institute of Parasitic Diseases. Written informed consent was obtained from the patients from the Burn Center of the Third People’s Hospital of Wuxi. *S. aureus* ATCC 29213 was used asa susceptible reference strain. The *S. aureus* strains were cultured aerobically in tryptic soy broth (TSB) (Difco Laboratories, Detroit, MI, United States).

### MRSA Identification

Methicillin-resistant *Staphylococcus aureus* identification was performed by polymerase chain reaction (PCR)-based amplification of the *mecA* gene according to a previously described method (He et al., 2017).

### Antibiotic Susceptibility Test

Minimal inhibitory concentrations (MICs) for TZ, erythromycin (EM), gentamicin (GM), levofloxacin (LE), oxacillin (OX), tetracycline (TC), and vancomycin (VA) against the *S. aureus* strains were evaluated according to the standard broth dilution method recommended by the Clinical Laboratory Standards Institute (CLSI) (Clinical and Laboratory Standards Institute [CLSI], 2009).

### Biofilm Formation Assays

A 96-well microtiter plate assay was carried out to evaluate biofilm formation ability as described previously (He et al., 2017). Briefly, the wells of a cell culture-treated plate were inoculated with approximately 2 × 10^4^ cells of *S. aureus* in 200 μl of TSB with different concentrations of TZ. After 24 h of static incubation under aerobic conditions at 37°C, the optical density (OD) of the supernatant in each well was measured at 600 nm. The biofilm that remained in the well was stained with 1% crystal violet (CV) solution and destained with 200 μl of 95% ethanol. The OD value of the ethanol solution was then measured at 590 nm. The biofilm formation index (BFI) was used to evaluate the biofilm formation ability. BFI = (OD_CV biofilm_ – OD_CV control_)/OD_planktonic_.

A 6-well microtiter plate assay was carried out to obtain biofilms for subsequent chemical analysis. Briefly, a cell culture-treated plate was inoculated with approximately1 × 10^6^ cells of *S. aureus* in 4 ml of TSB with a certain concentration of TZ. After 24 h of static incubation under aerobic conditions at 37°C, the supernatant was removed from each well, and the biofilm cells that remained were subjected to the following experiments.

A cover slip assay was carried out as described by Walker and Horswill (2012) for the preparation of biofilm samples for scanning electron microscopy (SEM). Briefly, culture-treated Thermanox cover slips15 mm in diameter (Nunc, Naperville, IL, United States) were placed in the wells of a 12-well plate. The wells with cover slips were inoculated with approximately 1 × 10^5^ bacterial cells in 2 ml of TSB with different concentrations of TZ. After 24 h of static incubation, the cover slip was kept at 4°C in fixative prior to microscopic observation.

### Biofilm Decomposition Assay

Biofilms were grown in a 96-well plateas described above. The biofilms were rinsed with PBS once and then treated with NaIO_4_ (10 mg/ml) in water for 2 h, proteinase K (100 μg/ml) in 20 mmol/l Tris-HCl (pH 7.5) with 100 mmol/l NaCl for 10 min, or DNase I (1000 U/ml) in 5 mmol/l MgCl_2_ for 12 h. The biofilms that remained were stained with CV and destained with 95% ethanol as described above. The percent difference in OD value was calculated and used to evaluate the biofilm detachment efficiency.

### Adhesion Assay

The ability of bacterial cells to attach to a polystyrene surface was determined according to a previous assay (He et al., 2017) with slight modification. Briefly, 1 ml of bacterial culture (18 h) was properly diluted to approximately 10^5^ CFU/ml and transferred to 24-well tissue culture-treated microplates. The inoculated microplates were aerobically incubated with or without TZ (16 μg/ml) at 37°C for 1 h. After removing the supernatant, the attached cells were collected, and enumerated using the spread plate technique on trypticase soy agar (TSA).

### Aggregation Assay

The bacterial aggregation ability was evaluated according to a method provided by Del Re et al. (2000) with slight modifications. The bacterial cells cultured with or without TZ (16 μg/ml) (18 h) were properly adjusted to approximately 10^8^ CFU/ml. The adjusted bacterial culture (3 ml) was transferred into a polystyrene test tube (12 mm × 75 mm) and mixed by gentle shaking. After mixing, 1 ml of the bacterial suspension was immediately transferred to determine the OD (A_0__h_) at 600 nm, and the remainder was incubated statically at 37°C for 2 h. After incubation, 1 ml of the upper suspension was transferred to determine the OD (A_2__h_). The aggregation efficiency was estimated as: Aggregation (%) = (1 – A_2__h_/A_0__h_) × 100.

### Transmission Electron Microscopy of MVs in Planktonic Cultures

Bacterial cells cultivated in TSB at 37°C for 18 h were collected at 1500 rpm and fixed with 2.5% glutaraldehyde, 2% paraformaldehyde, and 0.1% tannic acid in 0.1 M PBS (pH 7.4), followed by treatment with 1% OsO_4_. The samples were dehydrated by being passed through a series of graded concentrations of ethanol and acetone and then embedded in Epon epoxy resin. Ultrathin sections were obtained using an ultramicrotome with a diamond knife and examined under an electron microscope CM100 (Philips, Amsterdam, Holland) at 80 kV.

### Scanning Electron Microscopy of MVs in Biofilm Cultures

Biofilms developed on cover slips were dehydrated by being passed through a series of graded concentrations ofethanol (30, 50, 70, 80, 90, 95, and 100%) to remove water from the samples after glutaraldehyde fixation. After dehydration, the samples were dried with a critical point dryer, sputter coated with gold, and then examined under an electron microscope S-4800 (Hitachi, Minato-ku, Japan) at 15 kV.

### Chemical Analysis of Biofilms by FTIR

The biofilms formed in the wells of 6-well plates as described above were collected using a cell scraper and then lyophilized in a freezedryer. The biofilm powder was then subjected to Fourier transform infrared (FTIR) spectroscopic analysis by using a Varian670-IR spectrometer. To analyze the chemical constituents and their relative levels in the biofilms, the ordinate in the infrared spectrogram was normalized.

### RNA Isolation and Transcriptional Profiling

For RNA preparation, bacterial cells were grown in TSB for 12 h. The bacterial culture was mixed with RNAprotect Bacteria Reagent (Qiagen, Hilden, Germany) (1:2) to stabilize the RNA. Bacterial cells collected from the mixture were digested with 40 U/ml lysostaphin and 10 mg/ml lysozyme in Tris-EDTA buffer (pH 8.0).Total RNA was prepared using the RNeasy Mini Kit (Qiagen, Hilden, Germany) and quantified using a microvolume spectrophotometer. A reverse transcription kit (Qiagen, Hilden, Germany) was used to synthesize the cDNA. The oligonucleotide sequences presented in Table 1 were used as primers to target the conserved regions. Real-time reverse transcription PCR was performed in QuantiTect SYBR Green PCR master mix (Qiagen, Hilden, Germany) with the appropriate primers and by using the following conditions: 10 min at 95°C, followed by 40 cycles of 15 s at 95°C, 30 s at 55°C and 30 s at 72°C. The PCRs were performed on a LightCycler 480 PCR system (Roche, Penzberg, Germany).The cycle threshold (C_T_) value of the 16S ribosomal RNA gene was used to comparatively analyze the expression of the targeted genes.

**Table 1:** Sequences of oligonucleotide primers used for real-time RT-PCR.

Gene	Function	Primer direction: sequence	Reference
*16S rRNA*	Housekeeping gene	F: CGGTCCAGACTCCTACGGGAGGCAGCA	Wang et al., 2018
		R: GCGTGGACTACCAGGGTATCTAATCC	
*mecA*	Penicillin-binding protein 2A	F: GCAATCGCTAAAGAACTAAG	Fang and Hedin, 2003
		R: GGGACCAACATAACCTAATA	
*clfA*	Clumping factor A	F: ATTGGCGTGGCTTCAGTGCT	Atshan et al., 2012b
		R: CGTTTCTTCCGTAGTTGCATTTG	
*clfB*	Clumping factor B	F: ACATCAGTAATAGTAGGGGCAAC	Atshan et al., 2012b
		R: TTCGCACTGTTTGTGTTTGCAC	
*fnbA*	Fibronectin-binding protein A	F:CATAAATTGGGAGCAGCATCA	Atshan et al., 2012b
		R: ATCAGCAGCTGAATTCCCATT	
*fnbB*	Fibronectin-binding protein B	F: GTAACAGCTAATGGTCGAATTGATACT	Atshan et al., 2012b
		R: CAAGTTCGATAGGAGTACTATGTTC	
*icaA*	PIA/PNAG biosynthesis	F: ACACTTGCTGGCGCAGTCAA	Atshan et al., 2012b
		R: TCTGGAACCAACATCCAACA	
*icaD*	PIA/PNAG biosynthesis	F: ATGGTCAAGCCCAGACAGAG	Atshan et al., 2012b
		R: AGTATTTTCAATGTTTAAAGCAA	
*icaB*	PIA/PNAG biosynthesis	F: AGAATCGTGAAGTATAGAAAATT	Atshan et al., 2012b
		R: TCTAATCTTTTTCATGGAATCCGT	
*icaC*	PIA/PNAG biosynthesis	F: ATGGGACGGATTCCATGAAAAAGA	Atshan et al., 2012b
		R: TAATAAGCATTAATGTTCAATT	
*fabD*	Fatty acid biosynthesis	F: TTGACGCATAGTTCGGCATT	Wang et al., 2018
		R: ACTGCAGCCATGCTTCCTACA	
*fabF*	Fatty acid biosynthesis	F: TTCTGGTATCGGTGGTATGGA	Wang et al., 2018
		R: CTTGCCCAGTTGCCATATCA	
*fabG*	Fatty acid biosynthesis	F: GTTGCCGATGCTGATGAAGT	Wang et al., 2018
		R: TCATCCCACTCTTGTTCTTTCA	
*fabH*	Fatty acid biosynthesis	F: GATAACCGCACCTGCACCAT	Wang et al., 2018
		R: TGGATCAACTTGCAGCATGTT	
*fabI*	Fatty acid biosynthesis	F: GAAGACTTACGCGGACGCTT	Wang et al., 2018
		R: TGCTACCACCTTCTGGCATTA	
*sigB*	Sigma factor B	F: TGAAGATGCCAAGATTGCAGT	Wang et al., 2018
		R: CTAGGCCACCTTCGCGTAA	


*F, forward; R, reverse.*

### Statistical Analysis

All experiments were carried out in duplicate with three replicates. Statistical Analysis System (SAS Institute, Cary, NC, United States) software was used to analyze significant differences.

## Results

### The BWSA15 Isolate Was *mecA* Positive but Oxacillin Sensitive (OS-MRSA)

The *mecA* gene was detected in BWSA11 and BWSA15. The MIC values for the *S. aureus* type strain, BWSA11, BWSA15, and BWSA23 are listed in Table 2. The *S. aureus* strain BWSA11 was highly resistant to OX (MIC > 512 μg/ml) and other antibiotics. The *S. aureus* strains BWSA15 and BWSA23 were both sensitive to OX (MIC = 2 μg/ml).

**Table 2:** Minimum inhibitory concentration (μg/ml) of antibiotics against *S. aureus* strains.

Antibiotic (class)	ATCC 29213	BWSA11	BWSA15	BWSA23
TZ (beta-lactam)	16 (I)	>512 (R)	32 (R)	16 (I)
EM (macrolide)	0.5 (S)	>512 (R)	1 (I)	0.25 (S)
GM (aminoglycoside)	0.5 (S)	>512 (R)	1 (S)	0.5 (S)
LE (fluoroquinolone)	<0.25 (S)	128 (R)	0.5 (S)	<0.25 (S)
OX (beta-lactam)	0.25 (S)	>512 (R)	2 (S)	2 (S)
TC (tetracycline)	0.5 (S)	128 (R)	2 (S)	2 (S)
VA (glycopeptide)	0.5 (S)	1 (S)	0.5 (S)	1 (S)


*The abbreviations TZ, EM, GM, LE, OX, TC, and VA represent the antibiotics TZ, erythromycin, gentamicin, levofloxacin, oxacillin, tetracycline, and vancomycin, respectively. The antibiotic susceptibility of *S. aureus* was divided into antibiotic susceptible (S), intermediate (I) (if available), and resistant (R), with the resistance breakpoints for TZ as 8, 16, and 32 μg/ml, respectively; EM as ≤0.5, 1–4, and ≥8 μg/ml, respectively; GM as ≤4, 8, and ≥16 μg/ml, respectively; LE as ≤1, 2, and ≥4 μg/ml, respectively; OX as ≤2 and ≥4 μg/ml, respectively; TC as ≤4, 8, and ≥16 μg/ml, respectively; and VA as ≤2, 4, and ≥8 μg/ml, respectively.*

### Biofilm Formation inOS-MRSA15 Was Highly Induced by TZ

**FIGURE 1:**
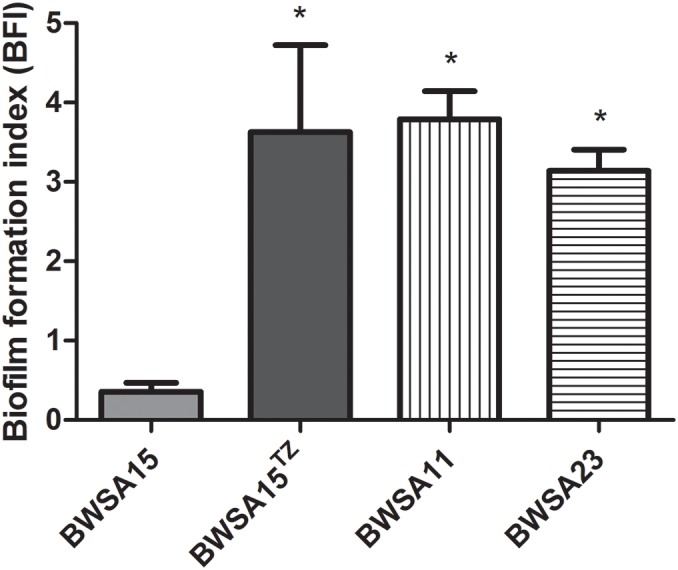
Relative biofilm formation index of *Staphylococcus aureus*. ^TZ^, in the presence of ceftazidime (TZ) (16 μg/ml). Error bars represent the standard deviations (*n* = 3). ^∗^*P* < 0.05 (Student’s *t*-test) indicate significant differences compared to BWSA15.

The biofilm formation index values are shown in Figure 1. In the absence of TZ, the highest BFI was achieved by BWSA11 (3.79) followed by BWSA23 (3.14), and BWSA15 (0.36), respectively. A significant increase in the biofilm forming abilities was observed in BWSA15 upon treatment of TZ at 32 μg/ml (4.88-fold), 16 μg/ml (1/4 MIC) (10.01-fold), and 8 μg/ml (5.03-fold) (*P* < 0.05). The biofilm forming abilities did not show significant increase in MSSA BWSA23 and OR-MRSA BWSA11 upon TZ treatment at any subinhibitory concentrations.

### Biofilm Formation in Both OS-MRSA 15 and OR-MRSA 11 Was Protein Dependent

The percent reduction in the biomass of the *S. aureus* biofilms is shown in Table 3. The main reduction in the biomass of the biofilms was caused by proteinase K for BWSA11 (81.88%) and BWSA15 (80.84%) and by NaIO_4_ for BWSA23 (83.16%).

**Table 3:** Percent reduction in the biomass of *S. aureus* biofilms treated with proteinase K (100 μg/ml), NaIO4 (10 mg/ ml), or DNase I (1000 U/ml).

Strain	Proteinase K	NaIO_4_	DNaseI
BWSA15	69.87 ± 7.90	17.73 ± 1.21	4.11 ± 0.83
BWSA15^TZ^	80.84 ± 5.53	5.47 ± 0.95	9.47 ± 1.76
BWSA11	81.88 ± 5.85	2.02 ± 0.66	7.03 ± 0.91
BWSA23	6.68 ± 0.95	83.16 ± 5.47	5.55 ± 0.64


*^*TZ*^The presence of TZ (16 μg/ml).*

### The Attachment Ability Was Not Significantly Enhanced in BWSA15 Upon Treatment With TZ

**FIGURE 2:**
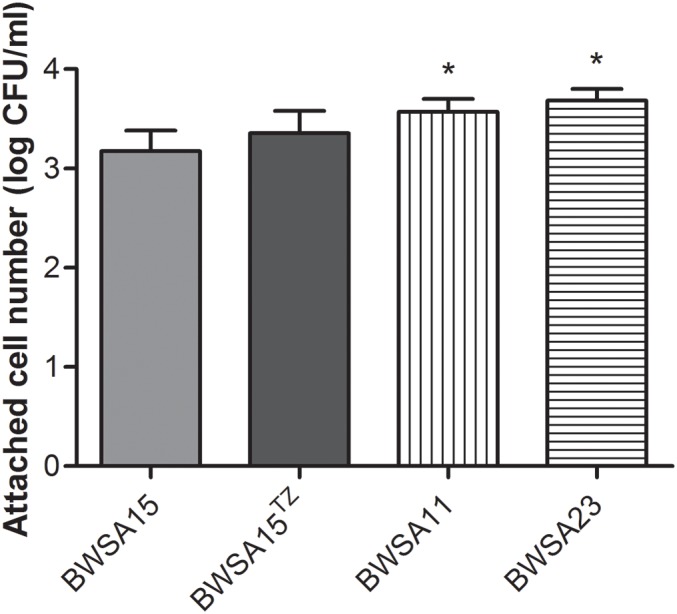
Surface adhesion of *S. aureus*. ^TZ^, in the presence of TZ (16 μg/ml). Error bars represent the standard deviations (*n* = 3). ^∗^*P* < 0.05 (Student’s *t*-test) indicate significant differences compared to BWSA15.

As shown in Figure 2, the highest attached cell number (log CFU/ml) was achieved by BWSA23 (3.68), followed by BWSA11 (3.57). The lowest attached cell number (log CFU/ml) was achieved by BWSA15 (3.17). There was no significant increase in the number of attached cells in BWSA15 upon treatment with TZ (16 μg/ml) (*P* < 0.05).

### The Aggregation Ability Was Significantly Enhanced in BWSA15 Upon Treatment With TZ

**FIGURE 3:**
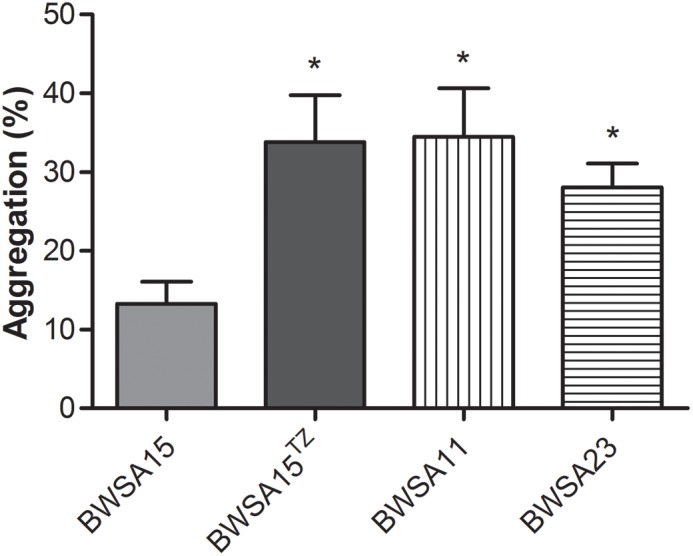
Intercellular aggregation of *S. aureus*. ^TZ^, in the presence of TZ (16 μg/ml). Error bars represent the standard deviations (*n* = 3). ^∗^*P* < 0.05 (Student’s *t*-test) indicate significant differences compared to BWSA15.

As shown in Figure 3, the highest aggregation ability (log CFU/ml) was observed inBWSA11 (34.48%), followed by BWSA15 in the presence of TZ (16 μg/ml) (33.82%). The lowest aggregation ability was observed in BWSA15 (13.26%). There was a significant increase in the percentage of aggregated cells in BWSA15 upon treatment with TZ (16 μg/ml) (*P* < 0.05).

### The Cell Junction in the Biofilm Formed by OS-MRSA15 in the Presence of TZ Was Mediated by Exposed MVs

**FIGURE 4:**
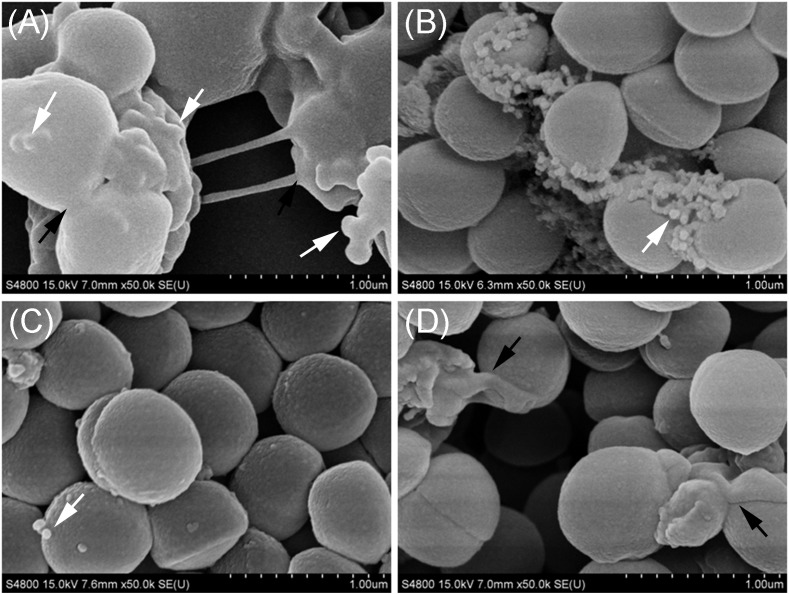
Scanning electron microscopy images of MVs formed by *S. aureus* BWSA15 **(A)**, *S. aureus* BWSA15 in the presence of TZ **(B)**, *S. aureus* BWSA11 **(C)**, and *S. aureus* BWSA23 **(D)**. ^TZ^, biofilm formed under TZ stress (16 μg/ml); white arrows indicate MVs; black arrows indicate mucous-like extracellular substances.

The membrane vesicles produced by *S. aureus* BWSA15 in the presence or absence of TZ were present in large numbers on the cell surfaces and formed intercellular aggregates (Figure 4A,B). Intercellular junctions mediated by MVs were observable in biofilms formed by *S. aureus* BWSA15 under different conditions. In contrast, few MVs were present on the cell surfaces of biofilm-forming *S. aureus* BWSA11 (Figure 4C), and even fewer MVs were observable in biofilms formed by *S. aureus* BWSA23 (Figure 4D). The intercellular junctions mediated by MVs were not visible in biofilms formed by either *S. aureus* BWSA11 or *S. aureus* BWSA23. The MVs produced by *S. aureus* BWSA15 in the absence of TZ were covered along with the matrix in a mucous-like extracellular substance, while the MVs produced by *S. aureus* BWSA15 in the presence of TZ or by *S. aureus* BWSA11 appeared exposed in the biofilms. In contrast to the intercellular junction of *S. aureus* BWSA15, that of biofilm-forming *S. aureus* BWSA23 was mediated by a polysaccharide-like substance instead of MVs.

### MVs From the Biofilm-Forming Cultures of OS-MRSA 15 Were Observable Intracellularly and Extracellularly

**FIGURE 5:**
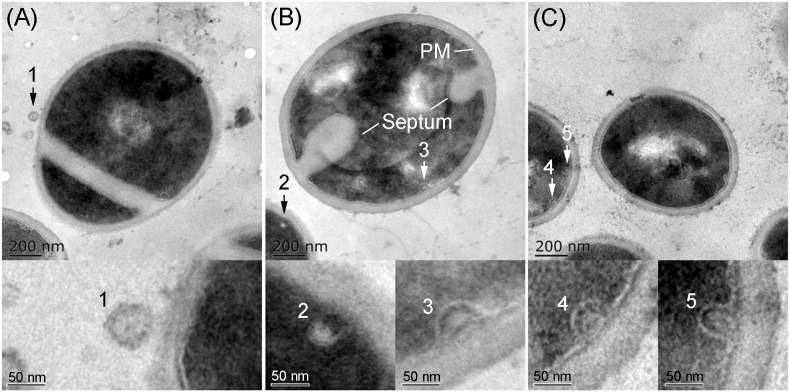
Secretion and production of MVs by *S. aureus*. TEM images of thin sections of biofilm-forming *S. aureus* BWSA15 in the presence of TZ **(A,B)** and *S. aureus* BWSA23 **(C)** showing the extracellular MVs (arrow 1) and intracellular MV-like structures (arrow 2–5). PM, plasma membrane.

TEM analysis showed that all *S. aureus* strains tested produced MVs during *in vitro* cultivation. These spherical and bilayered structures with diameters of approximately 50 nm were clearly visible on the surface of *S. aureus* or in the extracellular milieu of OS-MRSA (Figure 5A). The MV-like structures were also observed intracellularly and were characterized by fusion with plasma membranes (PMs) in all *S. aureus* strains tested (Figure 5B,C).

### The Levels of Carbohydrates and Ionized Carboxyl Groups in the Biofilm Matrix of OS-MRSA 15 Decreased Significantly Upon Treatment With TZ

**FIGURE 6:**
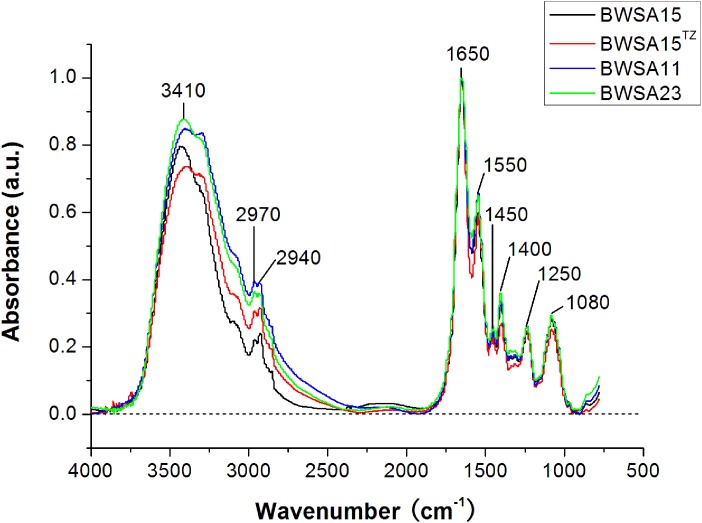
FTIR spectra of biofilms formed by BWSA15, BWSA11, and BWSA23. ^TZ^, in the presence of TZ (16 μg/ml).

The FTIR spectra of the biofilms and the corresponding functional group assignments are shown in Figure 6 and Table 4, respectively. The *S. aureus* strains BWSA15, BWSA11, and BWSA23 all exhibited the same characteristic absorption peaks in the infrared spectrum. For comparison and analysis, the absorbance values achieved by the C = O stretching were all normalized to 1.0. A significant decrease in the absorption of –OH bond stretching, C–O/C–O–C ring vibrations, and COO-vibrations was observed in BWSA15 in the presence of TZ. A significant increase in the absorption of C–H stretching was observed in BWSA15 in the presence of TZ.

**Table 4:** Main functional group assignments of infrared bands identified in *S. aureus* biofilms.

Frequency (cm^-1^)	Assignment	Reference
3410	Symmetric and asymmetric stretching of –OH bond in water	Ibrohim et al., 2006; Humbert and Quilès, 2011
2970	C–H asymmetric and symmetric stretching modes of methyl in fatty acids	Whittaker et al., 2003; Jiao et al., 2010; Humbert and Quilès, 2011
2940	C–H asymmetric and symmetric stretching modes of methylene in fatty acids	Whittaker et al., 2003; Jiao et al., 2010; Humbert and Quilès, 2011
1650	C = O stretching in amide I from proteins	Jiao et al., 2010; Humbert and Quilès, 2011; Probst et al., 2013
1550	N–H bending in amide II from proteins	Jiao et al., 2010; Humbert and Quilès, 2011; Probst et al., 2013
1450	C–H deformation of methylene in fatty acids	Humbert and Quilès, 2011; Probst et al., 2013
1400	Symmetric stretching vibration of COO^-^	Jiang et al., 2004; Humbert and Quilès, 2011
1250	Asymmetric stretching vibration of PO_2_^-^	Wong et al., 1991
1080	C–O and C–O–C ring vibrations in polysaccharides	Humbert and Quilès, 2011; Probst et al., 2013


### The Transcriptional Expression of Surface Adhesin-Associated Genes Was Downregulated in OS-MRSA 15 in the Presence of TZ

**FIGURE 7:**
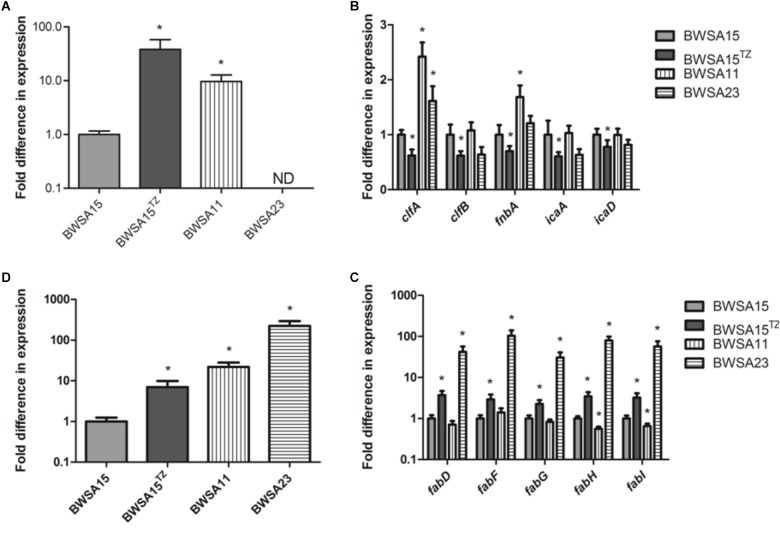
Fold change in the transcriptional expression of genes associated with **(A)** the biosynthesis of the PBP2a protein (*mecA*), **(B)** the surface adhesins including the surface proteins (*clfA, clfB*, and *fnbA*) and the exopolysaccharides PIA/PNAG (*icaA* and *icaD*), **(C)** the membrane fatty acids (*fabD, fabF, fabG*, and *fabH*), and **(D)** sigma factor B (*sigB*) in *S. aureus*. ND, not detectable. ^TZ^, in the presence of TZ (16 μg/ml). Error bars represent the standard deviations (*n* = 3). ^∗^*P* < 0.05 (Student’s *t*-test) indicate significant differences compared to BWSA15.

The gene expression levels of biofilm-forming *S. aureus* BWSA15 in the presence of TZ, BWSA11, and BWSA23 were all compared with those of BWSA15 and normalized. The relative gene expression patterns are shown in Figure 7. The expression levels of genes tested in BWSA15 were all normalized to 1. For methicillin resistance, the highest fold change was observed for the gene *mecA* in BWSA15 in the presence of TZ (37.90-fold), which exhibited a significant increase in the expression of this gene (*P* < 0.05).The expression of *mecA* was not detectable in *S. aureus* BWSA23 (Figure 7A). A significant decrease in the expression of clumping factor A (*clfA*) (0.62-fold) and clumping factor B (*clfB*) (0.64-fold) was observed in BWSA15 in the presence of TZ (*P* < 0.05) (Figure 7B). The lowest REI of *fnbA* was observed in BWSA15 (0.70),which exhibited a significant decrease in the expression of this gene in the presence of TZ (*P* < 0.05) (Figure 7B). For PIA/PNAG, a significant decrease in the expression of *icaA* and *icaD* was observed in BWSA15 in the presence of TZ (*P* < 0.05) (Figure 7B). The expression of *icaB* and *icaC* was not detectable by the primers used in this study. For membrane fatty acids, the highest expression levels of *fabD, fadF, fadG, fadH*, and *fadI* were observed in BWSA23, with fold increase values ranging from 30.44 to 103.33 (Figure 7C). A significant increase (2.27∼3.70-fold) in the expression of *fabD, fadF, fadG, fadH*, and *fadI* was observed in BWSA15 upon treatment with TZ (*P* < 0.05). For sigma factor B, the highest expression level of *sigB* was observed in BWSA23 (223.56-fold), followed by BWSA11 (21.91-fold) (Figure 7D). A significant increase (7.02-fold) in the expression of *sigB* was observed in BWSA15 in the presence of TZ (*P* < 0.05).

## Discussion

The biofilm formation activity of an OS-MRSA strain was investigated in the presence of a subinhibitory concentrations of TZ and compared with that of MSSA and OR-MRSA. To elucidate the functional roles of MSCRAMMs in the alteration of the biofilms of OS-MRSA under antibiotic stress, the transcriptional expression of PIA or PNAG, clumping factors A and B (ClfA and ClfB), collagen-binding protein, and fibronectin-binding factor A were investigated. To understand the relationship between MV biosynthesis and antibiotic stress response, fatty acids, sigma factor B and PM-associated proteins, which are vital for bacterial survival under antibiotic pressure, were transcriptionally investigated.

Given the observation regarding the polysaccharide-dependent decomposition characteristics of methicillin-sensitive BWSA23 (MSSA) and the protein-dependent decomposition characteristics of methicillin-resistant BWSA11 (OR-MRSA) and BWSA15 (OS-MRSA), there appears to be a significant difference in the mechanisms of biofilm formation between MSSA and MRSA. The gene *mecA* encodes the penicillin-binding protein 2a (PBP2a), which is involved in cell wall biosynthesis in MRSA. Given that the biofilms formed by BWSA 11 and BWSA15 under beta-lactam pressure were both characterized by the disappearance of polysaccharide-like exopolymeric substances, activation of the *mecA* gene may be directly, and indirectly involved in the downregulation of the production of PIA/PNAG during biofilm formation by MRSA. Similar results were also observed in other studies (Pozzi et al., 2012; Cortes et al., 2015). Since TZ did not trigger significant increase in biofilm forming ability in BWSA 11 and BWSA 23, the enhancement of the biofilm formation phenotype observed in BWSA15 upon treatment with a subinhibitory concentration of TZ is mainly attributed to increased aggregation ability caused by biological response rather than simple physical interaction during cell wall biosynthesis. OS-MRSA occurrence has been increasingly reported worldwide in recent years (Chen et al., 2012; Andrade-Figueiredo and Leal-Balbino, 2016). The heterogeneous antibiotic resistance and interrelated biofilm inducibility of OS-MRSA in response to beta-lactams pose a great challenge for chemotherapy of wound infections.

**FIGURE 8:**
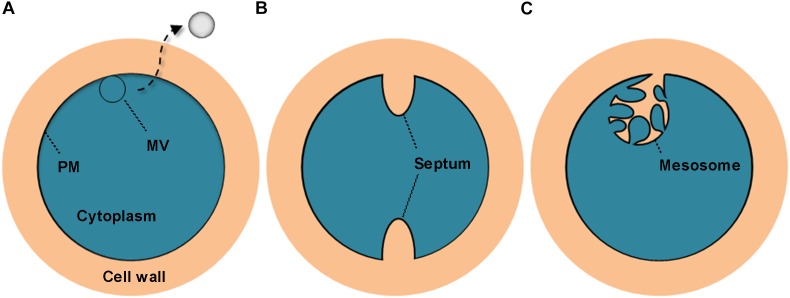
Schematic representation of the cross section of the MV precursor, septum, and mesosome in *S. aureus*. The MV precursor is characterized by fusion between the MV-like structure and the PM **(A)**; the septum is characterized by cell wall extension along with a non-coiled invagination in the PM **(B)**; and the mesosome is characterized by cell wall infiltration in a folded and coiled invagination in the PM **(C)**. PM, plasma membrane.

All *S. aureus* strains tested in this study were found to liberate MVs to the extracellular space. Similar findings were reported in other studies (Lee et al., 2009; Gurung et al., 2011). Notably, we observed that these bilayered, round or oval structures also exist intracellularly, fused with the PM. These PM-fused MV-like organelles are structurally different from the septum and the mesosome (Nanninga, 1971; Santhana Raj et al., 2007; Figure 8), which might provide a more intuitive view of the origin of MVs. In addition to *S. aureus* cells, MVs are the other substantial structural components of the biofilms formed by BWSA15, especially under TZ pressure, suggesting that MVs contribute mainly to biofilm formation by BWSA15, while few MVs were identified in the biofilms formed by BWSA11 and BWSA23. In terms of morphology, the appearance of intercellular MVs and the disappearance of polysaccharide-like extracellular substances from BWSA15 biofilms in response to TZ and the simultaneous thickening of biofilm-forming cells demonstrate that MVs play a crucial role in mediating intercellular interactions rather than simply participating in recruitment. The phenotypic alteration of biofilms formed by BWSA15 as well as the phenotypic difference between biofilms formed by BWSA15 and BWSA11 indicates the complexity of the regulatory mechanism of biofilm formation inMRSA.

The *icaABCD* operon encodes enzymes involved in the biosynthesis of PIA/PNAG. The genes *icaA, icaD, fnbA, fnbB*, and *clfA* are among the most prevalent genes in both MSSA and MRSA (Atshan et al., 2012b). Consistent with the phenotypes observed in BWSA15, the antibiotic-induced downregulation of *icaA* and *icaD* is mainly responsible for the disappearance of polysaccharide-like exopolymeric substances. We found that the differences in the transcriptional expression of *clfA, clfB*, and *fnbA* between BWSA11 and BWSA15 in the absence of antibiotic stress were significantly correlated with the differences in biofilm formation by these strains, suggesting the involvement of *mecA* in the regulation of the native expression of the surface adhesin proteins during biofilm formation by MRSA strains. However, given that we observed a significant decrease in the transcriptional expression of *clfA, clfB*, and *fnbA* in BWSA15 in response to TZ, it is evident that the biofilm formation in BWSA15 upon the treatment with subinhibitory concentration of TZ could not attributed to the expression of PIA/PNAG and the cell wall-anchored protein adhesins. However, the potential roles of certain virulence determinants delivered by *S. aureus* MVs in biofilm formation could not be excluded (Lee, 2012). The above results indicate the recruitment of different regulatory pathways induced by beta-lactams for *mecA*-dependent biofilm formation by BWSA15. These findings further strengthen the genetic heterogeneity of biofilm formation in MRSA.

It has been suggested that the increase in the levels of protein components relative to the levels of carbohydrates among exopolymeric substances increases bacterial hydrophobicity (Jorand et al., 1998). This physical property is closely associated with bacterial adhesion (Braga and Reggio, 1995). Given the significant increase in the total protein levels relative to carbohydrate levels in the biofilm matrix of BWSA15 in response to TZ, the primary non-specific adhesion of *S. aureus* appears to be due mainly to cell and cell surface hydrophobicity (Pascual et al., 1986). The proteins involved in the response to stress might contribute to the increase in the total protein levels relative to the carbohydrate levels in the biofilm matrix. Given the downregulation of the membrane-associated IcaA and IcaD of *S. aureus* BWSA15 in response to TZ, the *S. aureus* cells seem to reduce the functional levels of non-vital proteins in the PM, which could be due to the fitness cost created by sufficient redeployment of vital proteins such as PBP2a to cope with cell wall stress (Ender et al., 2004; Collins et al., 2010). This finding might also provide a reasonable explanation for the phenotypic alteration in biofilm formation by *S. aureus* in terms of the acquisition of methicillin resistance (O’Neill et al., 2007; Pozzi et al., 2012; McCarthy et al., 2015).

Fatty acids are another major component of the PM. The proper regulation of genes involved in fatty acid biosynthesis in *S. aureus* is crucial for the bacteria to cope with antibiotic stress (Wang et al., 2018). The increase in the transcription of *fabD, fabF, fabG, fabH*, and *fabI* in BWSA15 demonstrates the vital role of the proper expression of fatty acids in the maintenance of the cell viability of BWSA15 in response to antibiotics. The simultaneous occurrence of changes in transcriptional expression and genotypic changes in methicillin resistance as well as phenotypic changes in biofilm formation in BWSA15 indicate the involvement of fatty acids in biofilm formation by MRSA. However, the basal transcription of these fatty acid synthesis-associated genes did not vary consistently with the level of phenotypic biofilm formation or methicillin resistance between BWSA15 and BWSA11, indicating the heterogeneity in genetic origins among MRSA strains. In addition, the above mentioned increase in hydrophobicity is also reflected in the increase in the non-ionization of the carboxyl groups in the fatty acids in BWSA15 in response to TZ (Gross et al., 2001; Tribedi and Sil, 2014), which led to reduced repulsive force and therefore to consolidation of the hydrophobic interactions between the MVs and the bacterial cells, ultimately promoting biofilm formation by BWSA15.

Sigma B is a global regulatory factor that is involved in various stress responses (Pane-Farre et al., 2006). Similar to the observation reported by Wang et al. (2018), the activation of *fabD, fabF, fabG, fabH*, and *fabI* occurred simultaneously with the upregulation of *sigB*, suggesting the involvement of sigma B in the regulation of fatty acids. The expression of the *mecA* gene exhibited the same trend as that observed for *sigB* in BWSA15. However, it has been shown that *sigB* is not highly responsive to the *mecA*-dependent expression phenotype of methicillin resistance (Morikawa et al., 2001; Knobloch et al., 2005), even though this gene has been shown to have some effect on SCC*mec* excision (Zhang et al., 2016). As demonstrated in *Escherichia coli*, sigma E can be activated by the expression of outer membrane proteins (Hayden and Ades, 2008), and the activation of sigma B expression could possibly be triggered by the production of cytoplasmic PBP2a in *S. aureus*.

## Conclusion

In conclusion, there is an association between methicillin resistance and biofilm formation. The biofilms formed by OS-MRSA and OR-MRSA are both protein-dependent but significantly different in biofilm morphology and formation mechanisms. Beta-lactams can induce biofilm formation in OS-MRSA. MVs are not only the main structural components in addition to the cells in the biofilms formed by OS-MRSA under antibiotic stress but also crucial in mediating intercellular junctions. The phenotypic alteration of the biofilms formed by OS-MRSA strains was highly correlated with the upregulation of genes associated with cell wall biosynthesis (*mecA*), PM biosynthesis (fatty acid biosynthesis-associated genes), and sigma B, as well as with downregulation of MSCRAMMs (*icaA, icaD, clfA, clfB*, and *fnaA*). The functional roles of MVs in the development of biofilms formed by OS-MRSA are mainly associated with increased hydrophobicity, which can be achieved by an increase in protein levels relative to carbohydrate levels as well as by an increase in the levels of non-ionized carboxyl groups in fatty acids. This finding also provides new insights into the molecular association between methicillin resistance and biofilm formation.

## Author Contributions

XLH and FL contributed to the design of the study. XLH, FL, SL, YY, and JX contributed to the acquisition of the data. XQH, FL, YYY, TG, and YH contributed to the analysis of the data. All authors contributed to data interpretation, drafting the manuscript, critically revising the manuscript for important intellectual content, and approved the final version of the manuscript.

## Conflict of Interest Statement

The authors declare that the research was conducted in the absence of any commercial or financial relationships that could be construed as a potential conflict of interest.
